# Visual perception can account for the close relation between numerosity processing and computational fluency

**DOI:** 10.3389/fpsyg.2015.01364

**Published:** 2015-09-09

**Authors:** Xinlin Zhou, Wei Wei, Yiyun Zhang, Jiaxin Cui, Chuansheng Chen

**Affiliations:** ^1^State Key Laboratory of Cognitive Neuroscience and Learning, IDG/McGovern Institute for Brain Research, Siegler Center for Innovative Learning, Beijing Normal UniversityBeijing, China; ^2^Department of Psychology and Behavioral Sciences, Zhejiang UniversityHang Zhou, China; ^3^Department of Psychology and Social Behavior, University of California, IrvineIrvine, CA, USA

**Keywords:** visual perception, numerosity, computational fluency, arithmetic, approximate number system

## Abstract

Studies have shown that numerosity processing (e.g., comparison of numbers of dots in two dot arrays) is significantly correlated with arithmetic performance. Researchers have attributed this association to the fact that both tasks share magnitude processing. The current investigation tested an alternative hypothesis, which states that visual perceptual ability (as measured by a figure-matching task) can account for the close relation between numerosity processing and arithmetic performance (computational fluency). Four hundred and twenty four third- to fifth-grade children (220 boys and 204 girls, 8.0–11.0 years old; 120 third graders, 146 fourth graders, and 158 fifth graders) were recruited from two schools (one urban and one suburban) in Beijing, China. Six classes were randomly selected from each school, and all students in each selected class participated in the study. All children were given a series of cognitive and mathematical tests, including numerosity comparison, figure matching, forward verbal working memory, visual tracing, non-verbal matrices reasoning, mental rotation, choice reaction time, arithmetic tests and curriculum-based mathematical achievement test. Results showed that figure-matching ability had higher correlations with numerosity processing and computational fluency than did other cognitive factors (e.g., forward verbal working memory, visual tracing, non-verbal matrix reasoning, mental rotation, and choice reaction time). More important, hierarchical multiple regression showed that figure matching ability accounted for the well-established association between numerosity processing and computational fluency. In support of the visual perception hypothesis, the results suggest that visual perceptual ability, rather than magnitude processing, may be the shared component of numerosity processing and arithmetic performance.

## Introduction

Many previous studies have shown that numerosity processing (e.g., comparison of numbers of dots in two dot arrays) is significantly correlated with arithmetic performance (e.g., Halberda et al., 2008; Libertus et al., 2011; Lyons and Beilock, 2011). Researchers have attributed this association to the fact that both tasks share approximate number or magnitude processing (e.g., Halberda et al., 2008).

Some recent studies, however, have questioned the magnitude hypothesis (e.g., Inglis et al., 2011; Sasanguie et al., 2012, 2013; Vanbinst et al., 2012; Gilmore et al., 2013). In this paper, we are proposing an alternative hypothesis—the visual perception hypothesis. Because both numerosity processing and arithmetic performance are typically based on visual information, visual perceptual processing might be critical for both tasks and thus account for their association. Specifically, the current investigation tested the alternative hypothesis that visual perceptual ability (as measured by a figure-matching task) would account for the close relation between numerosity processing and arithmetic performance (computational fluency).

### Close relation between numerosity processing and arithmetic performance

The close relation between numerosity processing and arithmetic performance has been supported by several lines of research (Halberda et al., 2008; Libertus et al., 2011; Lyons and Beilock, 2011). First, children with developmental dyscalculia show severe impairment in numerosity judgment (e.g., Landerl et al., 2004; Geary et al., 2009; Piazza et al., 2010). Specifically, they had deficits in counting dots (Landerl et al., 2004; Butterworth, 2005) and numerosity comparison (Piazza et al., 2010). Second, numerosity processing has also been linked to individual differences in mathematical performance in typically developing children (e.g., Halberda et al., 2008, 2012; Mundy and Gilmore, 2009; Inglis et al., 2011; Libertus et al., 2011, 2013a; Mazzocco et al., 2011; Bonny and Lourenco, 2013). For example, using a non-symbolic quantity processing task (i.e., spatially intermixed blue and yellow dots were presented on a computer screen for 200 ms, and subjects had to judge which color of dots was more numerous), Halberda et al. (2008) found that 14-year-old children's performance on this task was correlated with scores on standardized mathematical achievement tests (Woodcock–Johnson revised calculation subtest and Test of Early Mathematics Ability). It should be noted, however, several studies have failed to find a significant association between numerosity processing and arithmetic performance (Rousselle and Noël, 2007; Holloway and Ansari, 2009; Price et al., 2012; Sasanguie et al., 2014). Third, training based on numerosity processing has been found to promote the development of symbolic arithmetic abilities (e.g., Park and Brannon, 2013; Hyde et al., 2014). It seems, as Gilmore and colleagues (Gilmore et al., 2007) argued, that young children rely on their numerosity ability to learn symbolic mathematics.

The close relation between numerosity processing and mathematics ability has traditionally been explained in terms of their shared numerical magnitude processing (e.g., Halberda et al., 2008). Both numerosity and mathematical tasks activate the same brain areas (i.e., bilateral intraparietal sulcus) (e.g., Ansari and Dhital, 2006; Izard et al., 2008). In addition to the magnitude processing hypothesis, Gilmore et al. (2013) has recently proposed the inhibitory control hypothesis. In their study, participants were first asked to name circles and squares in black and white colors, and then to provide the opposite name for each (i.e., say “circle” for square and “square” for circle). They found that individual variation in inhibitory control could account for the close relation of dot comparison and mathematical performance (Gilmore et al., 2013), suggesting that a domain-general or non-numerical factor (inhibition) may also account for the close relation between numerosity processing and mathematical performance.

### Studies that did not support the numerical magnitude hypothesis

Although the close relation between numerosity processing and arithmetic performance has been supported by several lines of research, several studies have failed to find a significant association between numerosity processing and arithmetic performance (Rousselle and Noël, 2007; Holloway and Ansari, 2009; Price et al., 2012; Sasanguie et al., 2014). The studies suggest that the magnitude processing underlying numerosity processing might not be critical for arithmetic performance.

Some studies also tested alternative explanations (e.g., Fuhs and McNeil, 2013; Gilmore et al., 2013). For example, Gilmore et al. (2013) found that individual variation in inhibitory control could account for the close relation between dot comparison and mathematical performance. They used an inhibition test (Korkman et al., 2007). Participants were firstly asked to name circles and squares in black and white colors, and then required to provide the opposite name for each (i.e., say “circle” for square and “square” for circle). The study suggests that there is a domain-general factor (i.e., inhibition control) other than quantity processing that could account for the close relation.

### The visual perception hypothesis

Here we propose that a non-numerical factor—visual perception—can account for the relation between numerosity processing and mathematical performance because both tasks are typically based on rapid visual information processing. Several lines of research have provided indirect evidence for this hypothesis.

First, visuospatial processing in general (e.g., Berg, 2008; Krajewski and Schneider, 2009; Simmons et al., 2012; Van Der Ven et al., 2013; see a review by Hubbard et al., 2005) and visual perception in particular (Rosner, 1973; Kurdek and Sinclair, 2001; Sigmundsson et al., 2010) have been shown to be important for mathematical processing. For example, Rosner (1973) showed that visual perception was correlated with arithmetic performance even after controlling for auditory perception. In a longitudinal study, Kurdek and Sinclair (2001) found that kindergarteners' visuomotor integration and verbal skills predicted their mathematical achievement in fourth grade. Sigmundsson et al. (2010) also found that, compared to age-matched controls, children with low mathematical performance were less sensitive to visually coherent motions (i.e., objects moving consistently, say, in the same direction, rather than randomly).

Second, previous research has shown perceptual properties of numerosity processing (e.g., Burr and Ross, 2008; Tibber et al., 2012). For example, Burr and Ross (2008) found that apparent numerosity was decreased by adaptation to large numbers of dots and increased by adaptation to small numbers, just like the adaption of other primary visual properties of a scene such as color, contrast, size and speed. Tibber et al. (2012) further showed that the density and numerosity estimates were derived from a common underlying metric.

Third, previous studies found that when numerosity (i.e., dot arrays) was presented for less than 300 ms, there was a consistent relation between numerosity processing and arithmetic performance (e.g., Halberda et al., 2008, 2012; Gebuis and Reynvoet, 2011; Lourenco et al., 2012; Wei et al., 2012b). However, when the presentation was longer than 300 ms, the results were not consistent, with some studies showing positive relations (e.g., Mundy and Gilmore, 2009; Lyons and Beilock, 2011; Bonny and Lourenco, 2013), while other studies did not (e.g., Price et al., 2012; Fuhs and McNeil, 2013; Kolkman et al., 2013; Sasanguie et al., 2014). In other words, the speed of visual perception seems critical for a close relation between numerosity processing and arithmetic performance.

Fourth, visual perception has been shown to be closely associated with language processing (e.g., Eden et al., 1996; Demb et al., 1998; Sperling et al., 2005; see a review by Vidyasagar and Pammer, 2010). In a functional brain imaging study, Eden et al. (1996) found that individuals with dyslexia showed abnormal processing of visual motion. Demb et al. (1998) also found that dyslexia was associated with an abnormality in the magnocellular pathway of the early visual system. Sperling et al. (2005) found that dyslexic children had deficits in visual perceptual signal-noise discrimination. These results from studies of visual perception and language processing are informative to our research because both language and mathematical tasks rely on rapid processing of artificial symbols (e.g., Arabic digits, letters).

### The current study

To directly test the visual perception hypothesis, we examined whether visual perceptual ability would account for the close relation between numerosity processing and computational fluency (Halberda et al., 2008; Libertus et al., 2011; Lyons and Beilock, 2011). A figure-matching task was used to assess visual perceptual ability because it has been used by previous studies of visual perception (Basso et al., 1985; Van Strien et al., 1989).

A battery of other cognitive tasks (including memory, intelligence, and spatial ability tests) was included as comparison tasks. In addition to computational fluency, we also included mathematical achievement as an outcome variable to see if the visual perceptual processing hypothesis applied specifically to computational fluency or more generally to arithmetic performance. Finally, previous studies have shown age or grade differences in the relation between numerosity processing and mathematical performance (e.g., Inglis et al., 2011), so the current study investigated whether the visual perception hypothesis would apply to all three grade levels (third to fifth) included in the current study.

## Method

### Participants

This study tested 424 third- to fifth-grade children (220 boys and 204 girls, 8.0–11.0 years old). Table 1 shows detailed demographic information by grade level. These children were recruited from 12 classes of two ordinary schools (one urban and one suburban) in Beijing, China. Six classes were randomly selected from each school, but all students in each selected class participated in the study. There were ~30–40 children per class. All participants were native Chinese speakers and had normal or corrected-to-normal eyesight.

**Table 1:** **Participants' information by grade level**.

**Grade**	**Number (Male, Female)**	**Mean age(*SD*)**
3	120 (58, 62)	8.89 (0.45)
4	146 (77, 69)	9.83 (0.41)
5	158 (85, 73)	10.89 (0.47)
Total	424 (220, 204)	9.96 (0.92)

The current investigation was the first part of an instructional reform plan in select primary schools in Fengtai County, Beijing, China. The plan aimed to improve children's learning by providing them with adaptive intervention programs based on their own cognitive abilities. The plan was sponsored by the research unit affiliated with the local Department of Education. Schools' participation was voluntary and school principals decided which grades and classes would participate in the plan. The students' parents completed written consent forms after they were told about the instructional reform plan in school. All students in the chosen classes participated. The research component of this study was approved by the National Key Laboratory of Cognitive Neuroscience and Learning at Beijing Normal University, the Department of Education of the Fengtai County of Beijing, and the principals of the schools.

### Procedure

The battery of tests were administered in two 45-min sessions, separated by 7~10 days. Tests were administered to students (one class at a time) in a computer classroom. Each class was monitored by six or seven experimenters (four to six children per experimenter) as well as the teacher of that class. Instructions were given and a practice session was completed before each formal test. The tasks were administered in the same order for all students. For all but two tests (verbal working memory and visual tracing), children indicated their responses by pressing one of two keys on a computer keyboard. For the verbal working memory test, they entered a series of digits after hearing them; and for the visual tracing test, they used the mouse to indicate the correct answer. Students' responses were automatically recorded and sent over the Internet to a server located in our laboratory at Beijing Normal University. All data were collected between December 1~15, 2011.

The practice session for each task consisted of either four or six trials similar to those used in the formal test. The computer provided the child with feedback on the screen after each practice trial. For all the tasks, feedback for correct responses was “Correct! Can you go faster?” and feedback for incorrect responses was “It is wrong. Try again.” Children could ask experimenters any questions they had during the practice session. After all children in a class had finished the practice session and had no more questions for the experimenters, the main experimenter said, “Start,” and the children pressed any key to begin the formal test.

### Tests

To control for potential confounding cognitive processes (e.g., Halberda et al., 2008; Wei et al., 2012a), we included five other cognitive tests that have been used in previous similar research (e.g., Halberda et al., 2008; Wei et al., 2012a). We included a test to assess visual tracing ability because poor oculo-motor coordination has been linked to reading disability (Groffman, 1994) and mathematical deficits (Fischer et al., 2008; Groffman, 2009). A basic reaction time task was used in order to control for the effect of manual response and mental processing speed (cf., Butterworth's (2003), “Dyscalculia Screener,” which included a reaction time task). Non-verbal matrix reasoning was used to measure basic intelligence or reasoning ability, which has been correlated with mathematical performance (e.g., Rohde and Thompson, 2007; Kyttälä and Lehto, 2008). The digit span and mental rotation tasks were used to control the unique contribution from verbal-numerical working memory and visuospatial working memory (Berg, 2008).

A total of eight tasks were used and they were computerized using Web-based applications in the “Online Experimental Psychological System (OPES)” (www.dweipsy.com/lattice). In addition, general mathematical achievement scores were obtained from the schools.

#### Numerosity comparison

**Figure 1:**
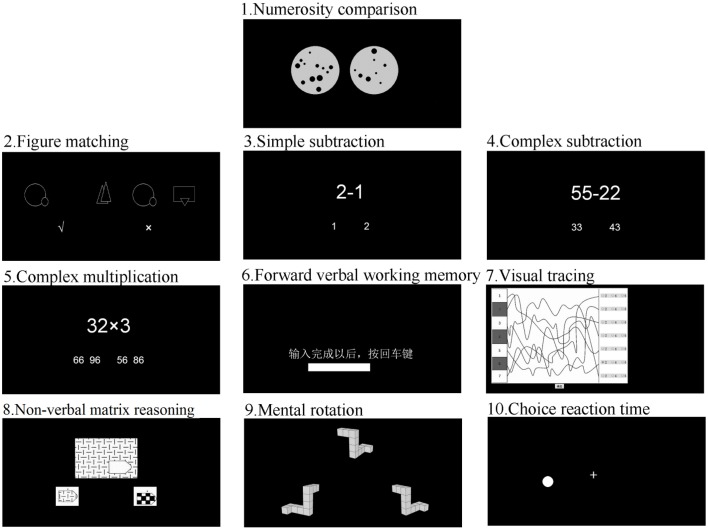
**Examples of stimuli for all cognitive tests**. Note: For the task forward verbal working memory, the Chinese words mean “After inputting answer, press key Enter.”

Two sets of dots were presented simultaneously on the screen, and participants were asked to judge which dot array contained more dots while ignoring the sizes of individual dots (see Figure 1). Participants pressed “Q” if they thought the array on the left contained more dots and “P” if they thought the array on the right contained more dots. The number of dots in each set varied from 5 to 32. The current investigation focused on the approximate number sense in numerosity processing, i.e., estimating the number of items (Feigenson et al., 2004). Therefore, we excluded the dot arrays within subitizing range (1–4 dots), which did not rely on estimation.

The two dot arrays for each trial were presented for 200 ms. After participants responded, there was a 1-s blank screen before the next trial. The test consisted of 120 trials. The dot arrays to be compared were created following a common procedure to control for continuous quantities in non-symbolic numerical discrimination (e.g., Halberda et al., 2008; Agrillo et al., 2013). For half of the trials, the total combined area of all dots in each set was controlled to be the same. For the other half of the trials, the average area of all dots in each set was controlled to be the same. The dots in a dot array were randomly distributed within a circle and the dots' sizes varied. The ratios for the two dot arrays ranged from 1.12 to 2.00. The trials were tested in three sessions, 40 trials for each session. Children were asked to complete all trials.

According to Gebuis and Reynvoet (2011), five visual properties of the numerosity comparison task should be considered: total surface area, envelope area or convex hull, item size, density (envelope area divided by total surface), and circumference. These properties were calculated for each of the 120 dot array pairs and were examined for their effects on task performance.

#### Figure matching

**Figure 2:**
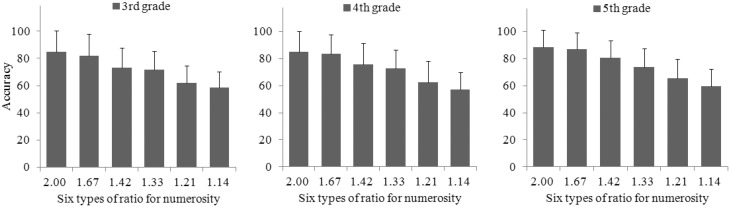
**The relations of performance on numerosity comparison (accuracy) for three grades and six types of ratios for numerosity (including 2.00, 1.67, 1.42, 1.33, 1.20, and 1.14)**.

The figure matching task was adapted from the identical picture test in the Manual for the Kit of Factor-Referenced Cognitive Tests (Ekstrom et al., 1976). There were 120 trials, each containing one target picture on the left side and three candidate pictures on the right side. The pictures were constructed from 150 abstract line figures. The four pictures were presented simultaneously for 400 ms. Participants were asked to judge whether the picture on the left side also appeared on the right side (see Figure 2), by pressing the button “Q” for yes or “P” for no. The 120 trials were grouped into three 40-trial sessions. Children were asked to complete all trials.

#### Arithmetic tests for computational fluency

There were three arithmetic tests: simple subtraction, complex subtraction, and complex multiplication. All were mental arithmetic tests; subjects were not allowed to use paper and pencil. The tests were similar to those used by Geary et al. for computational fluency (Geary et al., 2000). The simple subtraction and multi-digit multiplication tests were used in a previous study (Wei et al., 2012a). The problems for complex subtraction were selected from double-digit subtractions (e.g., 73–28).

##### Simple subtraction

For all 92 simple-subtraction problems (e.g., 6–2, 17–8), the minuends were 18 or smaller, and the differences were single-digit numbers. Two candidate answers were presented beneath each problem. Participants were asked to press the “Q” key to choose the answer on the left and the “P” key to choose the answer on the right. For this task, each incorrect candidate answer was within the range of the correct answer plus or minus 3 (i.e., ±1, ±2, or ±3). This was a time-limited (2 min) task.

##### Complex subtraction

All 96 problems in the complex subtraction task involved a double-digit number minus a double-digit number. Most problems required borrowing. Two candidate answers were presented beneath each problem: the correct answer and incorrect candidate answer (i.e., the correct answer ±1, ±10). The other aspects of the procedure (stimulus presentation, method of responding) were the same as those for the simple-subtraction task. This was a time-limited (2 min) task.

##### Complex multiplication

All 76 problems in the multiplication task involved one double-digit number multiplied by one single-digit number (e.g., 67 × 9). Every problem required carrying. Four candidate answers were presented beneath each problem: the correct answer and three incorrect candidate answers (i.e., the correct answer ±1, 10, and 100). The other aspects of the procedure (stimulus presentation, method of responding) were the same as those for the simple-subtraction task. This was a time-limited (2 min) task.

The numbers of correct trials for the three time-limited tasks were averaged to yield a composite score for computational fluency.

#### General mathematical achievement test

The local Department of Education developed and administered a general mathematical achievement test to all students in the county at the end of each semester. This test was curriculum-based and covered computation, mathematical concepts, and applied problem solving. Students had 90 min to complete the test.

#### Forward verbal working memory

The verbal working memory task was adapted from the digit span task of the Wechsler Intelligence Scale (Wechsler, 1974). Participants were presented a series of digits aurally through earphones. The length of sound for each digit was standardized to 200 ms. Participants were asked to remember the order of the digits and key them into the computer at the end of each series. The test began with 3 items (digits) and increased gradually until the children failed to key them correctly three times consecutively. The test lasted about 10 min.

#### Visual tracing

The task was adapted from Groffman's visual tracing test (Groffman, 1966). Several curved lines within a square interweaved with one another and started from the left side of the square and ended on the right side. Participants were asked to track a particular line from the beginning to the end only by eyeing (i.e., they were not allowed to use a finger or the cursor or an object to trace) and then to mark the correct end point by using the mouse. This task became more difficult as the total number of lines increased. There were 12 pictures, each used in 3 trials. This was a time-limited (4 min) task.

#### Non-verbal matrix reasoning

This test was adopted from the Raven's Progressive Matrices (Raven et al., 1998) to assess reasoning or general intelligence. First, we used two candidate answers rather than the original 4~6 candidate answers, because some younger children had difficulty using the mouse or choosing among 4 or 6 keys. Participants were asked to identify the missing segment of a figure according to the figure's inherent regularity. The participants were instructed to press “Q” with their left forefinger if the missing segment was on the left or “P” with their right forefinger if it was on the right. Second, due to the limited time allotted for this study, we had to shorten the task. The 80 items we used included 44 items from Standard Progressive Matrices (12 items from first set and 8 items from each one of other 4 sets) and 36 items from Advanced Progressive Matrices. The formal test was limited to 4 min. Similar shortened versions of this test have been used in previous studies (e.g., Bouma et al., 1996; Vigneau and Bors, 2001; Vigneau et al., 2006; Wei et al., 2012a). The shortened version had convergent validity as shown in its high correlation with a number series completion task that measures a type of reasoning in mathematics (Wei et al., 2012a).

#### Mental rotation

The mental rotation task was adapted from Vandenberg and Kuse (1978). The revised version had only two choices and allowed 3 min to complete. On each trial, one three-dimensional image was presented on the upper part of the screen, and two more were presented on the lower part of the screen. Participants were asked to choose which image from the bottom of the screen matched the image at the top; the matching image could be identified only by mental rotation. The non-matching image was a rotated mirror image of the target. The rotation angles of the matching images ranged from 15° to 345°, in intervals of 15°. On each trial, the stimuli remained on the screen until the participant responded by pressing the “Q” key to choose the image on the left and the “P” key to choose the image on the right. The mental rotation test consisted of 180 trials. This was a time-limited (3 min) test. The revised version had a high correlation with a figure analysis test, a task from the Cognitive Ability Test (level G, Lohman and Hagen, 2001).

#### Choice reaction time

On each trial of the choice reaction time test, a white dot was presented on a black screen, either to the left or to the right of a fixation cross. The position of the dot was within 15° of visual angle from the cross. Participants were asked to press the “Q” key if the dot appeared on the left and the “P” key if it appeared on the right. There were 30 trials in total (15 trials with the dot on the left and 15 trials with the dot on the right). Inter-stimulus intervals varied randomly between 1500 and 3000 ms.

Each of the tests described above had a practice session before the formal tests so that participants would be familiar with the procedure. During practice, the participants were given feedback (whether their answer was “Correct” or “Wrong”) and were encouraged to respond as quickly and accurately as possible. No feedback was provided during the formal tests.

For mental rotation, computational fluency, non-verbal matrix reasoning, and choice reaction time, split-half reliability ranged from 0.83 to 0.93 according to previous studies (Wei et al., 2012a,b). For figure matching, numerosity comparison, and visual tracing, split-half reliability was calculated from the data of the current study and it ranged from 0.86 to 0.96. For two tests—the mathematical achievement test developed by the local Department of Education and the verbal working memory (forward digit span) test, reliability statistics were not available. The local Department of Education did not formally report the psychometric statistics of their mathematical achievement test. The verbal working memory (forward digit span) test was almost the same as that used in the Wechsler Intelligence Scale (Wechsler, 1974).

### Data analyses

Following previous studies (e.g., Inglis and Gilmore, 2014), both accuracy and RT were reported for numerosity comparison and figure matching. For the four time-limited tests (i.e., computational fluency, mental rotation, visual tracing, and non-verbal matrices reasoning), we needed to control for the effect of guessing (e.g., Salthouse, 1994b; Salthouse and Meinz, 1995; Hedden and Yoon, 2006; Cirino, 2011). Guilford proposed a correction formula “S = R-W/ (n−1)” (S: the adjusted score, R: the number of correct responses, W: the number of incorrect responses. n: the number of alternative responses to each item) (Guilford, 1936). It has been used recently in studies of mathematical cognition (Cirino, 2011; Wei et al., 2012a,b) and cognition in general (Salthouse, 1994a; Putz et al., 2004; Hedden and Yoon, 2006). For the choice reaction time task, we calculated each participant's median reaction time and error rate. The gross mean error rate for the reaction time task was 4.2%, and was not further analyzed. For digit span, we used the longest series recalled and for general mathematical achievement, we used the total test scores reported by the schools.

Correlational analyses were used to investigate relations between the key measures of the study. Hierarchical multiple regression was then used to examine whether numerosity processing and figure matching made unique contributions to computational fluency and mathematical achievement. We conducted two sets of hierarchical multiple regression analyses. The first regression was conducted only to show the close relation between numerosity processing and computational fluency. General cognitive factors were first entered, followed by numerosity comparison. The second regression was conducted to show the role of visual processing in accounting for the above relation. General cognitive factors were first entered, followed by figure matching, and finally, numeroisty comparison.

## Results

The means and standard deviations of the cognitive tests and general mathematical achievement are displayed in Table 2. The intercorrelations among test scores are displayed in Table 3. As Table 3 shows, the correlation between figure matching and numerosity processing based on accuracy was consistently the highest among all the relations across all measures for each grade.

**Table 2:** **Means and standard deviations of test scores by grade level**.

**Task**	**Index**	**Mean(***SD***)**		
		**3rd grade**	**4th grade**	**5th grade**
Numerosity comparison	Percentage of correctness	72.04(9.9)	72.9(10.7)	75.9(9.7)
	Reaction time (millisecond)	612(162)	570(140)	581(118)
Figure matching	Percentage of correctness	67.3(11.0)	70.1(13.9)	72.3(10.6)
	Reaction time (millisecond)	1008(290)	965(253)	938(180)
Computational fluency	Adj. No. of correct trials	26.2(4.6)	28.8(5.1)	30.5(5.7)
Verbal WM forward	No. of recalled digits	7.6(1.2)	8.1(1.3)	8.2(1.3)
Visual tracing	Adj. No. of correct trials	12.5(5.6)	16.4(4.6)	17.2(5.4)
Non-verbal matrix reasoning	Adj. No. of correct trials	19.4(5.7)	18.7(6.3)	18.9(5.9)
Mental rotation	Adj. No. of correct trials	17.9(8.2)	20.7(7.7)	20.9(7.8)
Choice reaction time	Reaction time (millisecond)	433(133)	423(126)	421(152)
Mathematical achievement	Score(0–100)	87.9(6.2)	90.3(6.3)	83.5(10.6)

*Adj. No. of correct trials = Total correct trials minus total incorrect trials. This adjustment was to control for the effect of guessing in multiple choice tests*.

**Table 3:** **Intercorrelations among test scores by grade level**.

	**Test**	**1**	**2**	**3**	**4**	**5**	**6**	**7**	**8**	**9**	**10**
3rd grade	1. Numerosity comparison(ACC)	–									
	2. Nuemrosity comparison(RT)	0.65^***^	–								
	3. Figure-matching(ACC)	0.61^***^	0.35^***^	–							
	4. Figure-mathcing(RT)	0.49^***^	0.58^***^	0.53^***^	–						
	5. Computational fluency	0.37^***^	0.21^*^	0.47^***^	0.25^**^	–					
	6. Verbal WM forward	0.07	0.05	0.21^*^	0.19^*^	0.30^**^	–				
	7. Visual tracing	−0.00	−0.03	0.13	0.02	0.18^*^	0.28^**^	–			
	8. Non-verbal matrix reasoning	0.12	−0.02	0.33^***^	0.00	0.25^**^	0.10	0.25^**^	–		
	9. Mental rotation	−0.13	−0.14	0.10	−0.02	0.14	0.19^*^	0.16	0.08	–	
	10. Choice reaction time	−0.17	0.24^*^	−0.26^**^	−0.02	−0.10	−0.03	−0.09	−0.07	−0.06	–
	11. Mathematical achievement	0.28^**^	0.24^**^	0.32^***^	0.28^**^	0.36^***^	0.23^*^	0.09	0.16	−0.09	−0.09
4th grade	1. Numerosity comparison(ACC)	–									
	2. Nuemrosity comparison(RT)	0.65^***^	–								
	3. Figure-matching(ACC)	0.40^***^	0.37^***^	–							
	4. Figure-mathcing(RT)	0.41^***^	0.59^***^	0.41^***^	–						
	5. Computational fluency	0.29^***^	0.18^*^	0.34^***^	0.13	–					
	6. Verbal WM forward	0.07	−0.02	0.14	−0.05	0.24^**^	–				
	7. Visual tracing	0.07	0.03	0.03	−0.10	0.20^*^	0.06	–			
	8. Non-verbal matrix reasoning	0.24^**^	0.13	0.15	0.11	0.28^**^	0.02	0.19^*^	–		
	9. Mental rotation	0.07	0.11	−0.07	0.03	0.07	−0.03	0.20^*^	0.22^*^	–	
	10. Choice reaction time	0.02	0.07	−0.04	0.05	−0.12	−0.08	−0.15	−0.03	0.05	–
	11. Mathematical achievement	0.18^*^	0.03	0.14	−0.02	0.38^***^	0.20^*^	0.14	0.29^***^	0.19^*^	−0.18^*^
5th grade	1. Numerosity comparison(ACC)	–									
	2. Nuemrosity comparison(RT)	0.50^***^	–								
	3. Figure-matching(ACC)	0.53^**^	0.39^***^	–							
	4. Figure-mathcing(RT)	0.38^***^	0.50^***^	0.49^***^	–						
	5. Computational fluency	0.22^**^	0.06	0.36^***^	0.18^*^	–					
	6. Verbal WM forward	0.21^**^	−0.002	0.16^*^	0.08	0.08	–				
	7. Visual tracing	0.24^*^	−0.06	0.24^**^	0.02	0.19^*^	0.13	–			
	8. Non-verbal matrix reasoning	0.27^**^	0.15	0.19^*^	0.17^*^	0.20^*^	0.11	0.34^***^	–		
	9. Mental rotation	0.21^**^	0.13	0.27^**^	0.09	0.17^*^	0.08	0.34^***^	0.42^***^	–	
	10.Choice reaction time	−0.22^**^	0.02^*^	−0.12	0.05	−0.13	−0.15	−0.23^**^	−0.13	−0.07	–
	11. Mathematical achievement	0.25^**^	0.06	0.21^**^	0.12	0.43^***^	0.26^**^	0.33^***^	0.34^***^	0.27^**^	−0.22^**^

The sample size was 120 for 3th grade, 146 for 4th grade, and 158 for 5th grade,

* p < 0.05,

** p < 0.01,

*** *p < 0.001*.

*RT, Reaction time; ACC, Accuracy (%)*.

The accuracy on the numerosity comparison test was ratio-dependent (see Figure 2), that is, the larger the ratio was between the larger and the smaller dot arrays, the higher the accuracy was, *r*_(6)_ = 0.94, *p* < 0.01 for third grade, *r*_(6)_ = 0.90, *p* < 0.05 for fourth grade, and *r*_(6)_ = 0.92, *p* < 0.01 for fifth grade. Even after considering the five visual properties of the dot arrays (total surface area, envelope area or convex hull, item size, density [envelope area divided by total surface], and circumference, Gebuis and Reynvoet, 2011), the accuracy across all trials (i.e., 120 dot array pairs) was still ratio-dependent, *r*_(113)_ = 0.31, *p* < 0.001.

**Figure 3:**
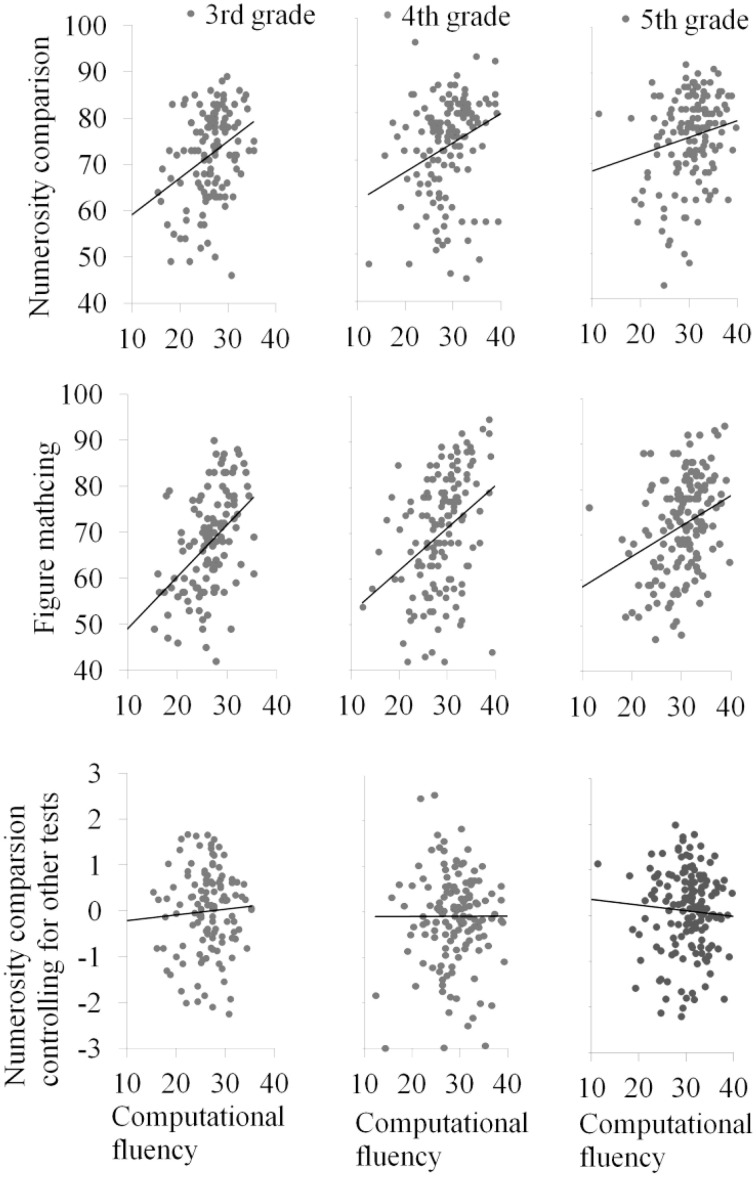
**Scatter plot of numerosity comparison and figure matching on computational fluency**.

Figure 3 shows scatter plots of numerosity comparison by computational fluency (top panel), figure matching by computational fluency (middle panel), and numerisoty comparison by computational fluency after controlling for figure matching and all other cognitive measures (bottom panel).

The results for the hierarchical multiple regression model are displayed in Table 4. Figure matching was a significant predictor of computational fluency after controlling for general cognitive processes (i.e., non-verbal matrix reasoning, visual tracing, mental rotation, choice reaction time, and verbal working memory) (Step 2) for all three grades. The regression coefficient for figure matching predicting mathematical achievement was not significant for any of the grades. After numerosity comparison was entered into the model (Step 3), figure matching remained as a significant predictor of computational fluency. Most relevant to our hypothesis, the relation between numerosity and computation was no longer significant after figure matching ability was added as a predictor. Figure matching ability accounted for a unique portion of the variance of computational fluency. Neither numerosity processing nor figure matching ability was significant in the regression equations for general mathematical achievement.

**Table 4:** **Results from hierarchical multiple regression analyses for the relations of numerosity processing and mathematical performance**.

**Grade**	**Predictors**	**Computational fluency**			**Mathematical achievement**		
		**Step 1**	**Step 2**	**Step 3**	**Step 1**	**Step 2**	**Step 3**
		***B (SE)***	***B (SE)***	***B (SE)***	***B (SE)***	***B (SE)***	***B (SE)***
3	Non-verbal matrix reasoning	0.16 (0.07)^*^	0.13 (0.07)	0.07 (0.07)	0.15 (0.10)	0.13 (0.10)	0.10 (0.11)
	Mental rotation	0.04 (0.05)	0.07 (0.05)	0.05 (0.05)	−0.11 (0.07)	−0.08 (00.7)	−0.10 (0.07)
	Visual tracing	0.04 (0.08)	0.05 (0.07)	0.05 (0.07)	0.01 (0.11)	0.01 (0.10)	0.01 (0.10)
	Choice reaction time	−0.00 (0.00)	0.00 (0.00)	0.001 (0.002)	−0.00 (0.00)	−0.00 (0.00)	−0.00 (0.00)
	Verbal WM forward	0.95 (0.35)^**^	0.83 (0.33)^*^	0.72 (0.33)^*^	10.27 (0.49)^*^	1.14 (0.48)^*^	0.99 (0.48)^*^
	Numerosity comparison(ACC)	–	0.16 (0.06)^**^	0.09 (0.06)	–	0.07 (0.08)	0.02 (0.09)
	Numerosity comparison(RT)	–	0.00 (0.00)	0.00 (0.00)	–	0.01 (0.01)	0.00 (0.01)
	Figure matching(ACC)	–	–	0.12 (0.05)^*^	–	–	0.08 (0.08)
	Figure matching(RT)	–	–	0.00 (0.00)	–	–	0.00 (0.00)
		*R*^2^ = 0.15^**^	Δ*R*^2^ = 0.11^***^	Δ*R*^2^ = 0.04	*R*^2^ = 0.10^*^	Δ*R*^2^ = 0.06^*^	Δ*R*^2^ = 0.02
4	Non-verbal matrix reasoning	0.20 (0.06)^**^	0.16 (0.07)^*^	0.15 (0.06)^*^	0.24 (0.08)^**^	0.21 (0.08)^*^	0.21(0.08)^*^
	Mental rotation	−0.00 (0.05)	−0.01 (0.05)	0.01 (0.05)	0.12 (0.07)	0.12 (0.07)	0.13 (0.07)
	Visual tracing	0.14 (0.09)	0.14 (0.09)	0.14 (0.09)	0.05 (0.11)	0.04 (0.11)	0.03 (0.11)
	Choice reaction time	−0.00 (0.00)	−0.00 (0.00)	−0.00 (0.00)	−0.00 (0.00)	−0.00 (0.00)	−0.00 (0.00)
	Verbal WM forward	0.85 (0.31)^**^	0.80 (0.30)^*^	0.69 (0.30)^*^	0.90 (0.38)^*^	0.83 (0.38)^*^	0.77 (0.38)^*^
	Numerosity comparison(ACC)	–	0.10 (0.05)^*^	0.07 (0.05)	–	0.11 (0.06)	0.10 (0.06)
	Numerosity comparison(RT)	–	0.00 (0.00)	−0.00 (0.00)	–	−0.01 (0.01)	−0.01 (0.01)
	Figure matching(ACC)	–	–	0.08 (0.03)^**^	–	–	0.04 (0.04)
	Figure matching(RT)	–	–	0.00 (0.00)	–	–	−0.00 (0.00)
		*R*^2^ = 0.16^***^	Δ*R*^2^ = 0.05^*^	Δ*R*^2^ = 0.04^*^	*R*^2^ = 0.17^***^	Δ*R*^2^ = 0.02	Δ*R*^2^ = 0.01
5	Non-verbal matrix reasoning	0.11 (0.09)	0.09 (0.09)	0.10 (0.08)	0.37 (0.15)^*^	0.35 (0.15)^*^	0.34 (0.15)
	Mental rotation	0.06 (0.07)	0.05 (0.07)	0.02 (0.06)	0.14 (0.11)	0.12 (0.11)	0.12 (0.11)
	Visual tracing	0.11 (0.09)	0.09 (0.09)	0.05 (0.09)	0.35 (0.16)^*^	0.34 (0.16)^*^	0.33 (0.16)
	Choice reaction time	−0.00 (0.00)	−0.00 (0.00)	−0.00 (0.00)	−0.01 (0.01)	−0.01 (0.01)	−0.01 (0.01)
	Verbal WM forward	0.15 (0.35)	0.05 (0.35)	−0.04 (0.34)	10.51 (0.60)^*^	1.42 (0.60)^*^	1.39 (0.61)
	Numerosity comparison(ACC)	–	0.09 (0.06)^*^	0.02 (0.06)	–	0.08 (0.10)	0.07 (0.11)
	Numerosity comparison(RT)	–	0.00 (0.01)	−0.01 (0.01)	–	0.00 (0.01)	0.00 (0.01)
	Figure matching(ACC)	–	–	0.17 (0.05)^**^	–	–	0.03 (0.10)
	Figure matching(RT)	–	–	0.00 (0.00)	–	–	0.00 (0.01)
		*R*^2^ = 0.07^*^	Δ*R*^2^ = 0.02	Δ*R*^2^ = 0.07^**^	*R*^2^ = 0.23^***^	Δ*R*^2^ = 0.01	Δ*R*^2^ = 0.002

* p < 0.05,

** p < 0.01,

*** *p < 0.001*.

*RT, Reaction time; ACC, Accuracy*.

## Discussion

The current study aimed to investigate the cognitive mechanism underlying the close relation between numerosity processing and arithmetic performance reported in previous studies. We first replicated the previous finding of a strong relation between numerosity processing and arithmetic performance. We further found that figure matching was more closely associated with numerosity comparison than were other types of cognitive processing (e.g., visual tracing, non-verbal matrices reasoning, and mental rotation). Further analyses showed that visual perception (figure matching) accounted for the association between numerosity processing and computational fluency. In contrast, neither numerosity processing nor visual perception was important for general mathematical achievement for fourth and fifth graders. As it has been argued elsewhere (Inglis et al., 2011), general mathematical achievement does not seem to depend on numerosity processing. Instead, general cognitive abilities such as verbal working memory are important for general mathematics achievement.

### Visual perception and computational fluency

Our results provide an alternative explanation of the well-established finding of a close association between numerosity processing (e.g., comparison of numbers of dots in two dot arrays) and computational fluency. That is, visual perceptual ability as measured by a rapid figure matching task, rather than the processing of non-symbolic numerical magnitude, may be the mechanism underlying that close relation. Indeed, if non-symbolic magnitude processing or the number sense were the mechanism for the close relation between numerosity comparison and computational fluency (e.g., Halberda et al., 2008), figure matching should not have fully accounted for the above relation because numerosity comparison involved the number sense but figure matching did not.

The importance of visual processing for computational fluency does seem obvious given the nature of the tasks involved. In our study, computational fluency was measured with a test similar to that used by Geary et al. (2000). It was a visually presented mental calculation test. According to Dehaene and Cohen's triple code model (Dehaene and Cohen, 1995, 1997), visually-presented arithmetic problems would involve visual codes (or Arabic number forms). Brain imaging studies have also demonstrated that arithmetic problems typically activate the parietal cortex (e.g., Rosenberg-Lee et al., 2011), which is believed to support visuospatial processing (e.g., Corbetta et al., 1998; Linden et al., 2003).

Another common aspect of the figure matching and numerosity comparison tasks was that both tasks involved brief presentation of the stimuli, so the speed of visual perception might be critical. Participants needed to quickly encode sensory inputs, access and retrieve information from long-time memory, and integrate different items of information in working memory. Indeed, we found that the reaction times for numerosity comparison and figure matching were generally correlated with computational fluency. Nevertheless, the correlations for the accuracy were even stronger, which was line with a similar finding by Halberda et al. (2012) that ANS precision indexed by Weber fraction (accuracy) had stronger correlations with school mathematics ability than did ANS precision indexed by reaction time. One possible reason for the stronger relation for accuracy than for RT is the brief presentation of the stimuli for both tasks (200 ms for numerosity comparison and 400 ms for figure matching in the current study; 200 ms for numerosity comparison in Halberda et al., 2008). With brief presentation, accuracy may better reflect visual perceptual ability than RT.

If visual perception is the mechanism behind the association between numerosity and computational fluency, we need to explain why some seemingly visual tasks did not account for that. First, we found that this relation was not accounted for by the following tasks that involved visual perception: visual tracing, non-verbal matrix reasoning, and mental rotation. One possible reason is that the speed of visual perception is not critical for these tasks because these tasks seem to be based on “slow form perception”: That is, participants tend to carefully analyze the “forms” involved in these time-consuming tasks. Specifically, they take time to make oculo-motor coordination for visual tracing (Groffman, 1966), to find rules for non-verbal matrix reasoning, and to mentally rotate images for mental rotation. Second, the choice reaction time task was also a visual task and furthermore it involved fast form perception, but it still did not account for the association between numerosity comparison and computational fluency. We think that the “form” in this task is minimal because it involves just a dot to the left or right of the fixation sign. Therefore, the choice reaction time probably involves little form processing. Of course, the above speculations need to be directly tested in future research by manipulating systematically the form complexity and the speed of form perception.

It is also worth pointing out that the relation between visual perception and computational fluency was consistent across all three grades for simple subtraction, but was significant only for higher grade levels for complex subtraction and complex multiplication. Children in lower grade were not very fluent at complex arithmetic because they had just started learning complex multiplication at the third grade (the lowest grade in the current investigation). Therefore, the speed of visual perception might not play an important role in their arithmetic performance.

### Explaining previous findings that supported the numerical magnitude hypothesis

On the surface, our results seem to be inconsistent with Halberda et al.'s finding (2008) that the close relation between numerosity processing and arithmetic performance remained after controlling for a large number of cognitive measures including verbal IQ, performance IQ, visual working memory, visual reasoning, spatial reasoning, reading, executive functions, and rapid lexical access. However, other than numerosity processing, none of the other cognitive tasks used in Halberda et al. study were rapidly presented, which would have explained why numerosity processing was found to remain a significant predictor of arithmetic performance in their study.

Other studies that supported the magnitude processing hypothesis are also worth a second look. For example, several studies found that the ANS was associated with early mathematics performance measured with the TEMA (Test of Early Mathematics Ability, Ginsburg and Baroody, 1983) in preschoolers (e.g., Halberda et al., 2008; Libertus et al., 2011, 2013a,b; Mazzocco et al., 2011; Bonny and Lourenco, 2013; Chu et al., 2013; Fuhs and McNeil, 2013; Starr et al., 2013). Interestingly, the majority of items of the TEMA task are not timed so children can have as much time as they need to answer the questions. Even more importantly, the majority of them have no visual forms. The fact that the ANS is associated with the TEMA that involves no visual forms and has no time limit seems to challenge the visual perception hypothesis. One explanation of these results is that these studied did not control for enough cognitive factors. For example, Bonny and Lourenco (2013) only controlled for receptive vocabulary. Libertus et al. (2011) controlled for vocabulary size (measured according to parents' judgment on words that they had ever heard their children speaking). Chu et al. (2013) controlled for intelligence, executive functions, and letter identification. In the study by Libertus et al. (2011), none of the following variables was controlled: overall intelligence, information-processing speed, working memory, or other cognitive abilities. The uncontrolled factors such as attention, working memory, processing speed, executive functions (e.g., inhibitory control), or general intelligence might be the reason for the relation between young children's TEMA score and ANS ability found in previous studies. As mentioned earlier, just one of the above factors (inhibitory control) could have accounted for the relation of ANS and preschoolers' math ability measured with TEMA (Fuhs and McNeil, 2013). Indeed, a number of studies have found that numerosity processing is not associated with mathematical performance, especially when confounding variables are controlled for (Holloway and Ansari, 2009; Soltész et al., 2010; Castronovo and Göbel, 2012; Sasanguie et al., 2012; Vanbinst et al., 2012; Fuhs and McNeil, 2013; Kolkman et al., 2013; Price and Ansari, 2013). For example, in a recent study of the relation between numerosity processing (comparison of 1–9 dots) and curriculum-based standardized mathematics achievement (including number knowledge, understanding of operations, simple arithmetic, word problem solving, measurement, and geometry) kindergarteners and first, second, and sixth graders, Sasanguie et al. (2012) found no significant correlations between non-symbolic number comparison and mathematics achievement. One explanation of the conflicting findings may be the way mathematical performance has been measured, either as computational fluency or as general mathematical performance. A closer examination of the literature shows that numerosity processing appeared to be associated with computational fluency (e.g., Halberda et al., 2008; Gebuis and Reynvoet, 2011; Inglis et al., 2011; Lyons and Beilock, 2011; Wei et al., 2012b), but not with general mathematical achievement (e.g., Göbel and Snowling, 2010; Inglis et al., 2011; Sasanguie et al., 2012, 2014; Vanbinst et al., 2012) or mathematical reasoning (Inglis et al., 2011; Wei et al., 2012b). The tests of general mathematical achievement used in previous studies covered a wider range of mathematical abilities, such as quantitative concepts, applied problem solving, number series completion, geometry, and so on (e.g., Inglis et al., 2011; Sasanguie et al., 2012, 2013; Vanbinst et al., 2012).

### Other factors of potential relevance

Gilmore et al. (2013) also found that individual variation in inhibitory control could account for the close relation between dot comparison and mathematical performance. The inhibitory control hypothesis is not incompatible with the visual perception hypothesis because visual perception might have been involved in the inhibition task. Participants had to retrieve other forms (e.g., square) based on the current form (e.g., circle), for which visual perception is important. Future research should contrast the two perspectives directly.

Although our discussion focused on the relation between numerosity processing and computational fluency in the visual modality, several studies have explored how people process numerosity in non-visual modalities (e.g., auditory, tactile) (e.g., Lipton and Spelke, 2003; Riggs et al., 2006; Tokita et al., 2013). To our knowledge, however, none of them have examined the relation between non-visual numerosity processing and computational fluency. According to the visual perception hypothesis, such a relation should not exist because visual perception is not directly involved. Future study should examine this speculation.

### Implications

Our results have important implications for our understanding of mathematical cognition and potential interventions for mathematical difficulties. They suggest that previous studies might have overemphasized the role of non-symbolic numerical quantity processing (or the number sense, the approximate number system) in the development of mathematical skills (e.g., Landerl et al., 2004; Halberda et al., 2008; Piazza et al., 2010). Previous studies also have emphasized non-symbolic quantity processing as a target of effective interventions for mathematical learning difficulty (e.g., Wilson et al., 2006; Butterworth and Laurillard, 2010; Park and Brannon, 2013; Hyde et al., 2014). Our results suggest that arithmetic intervention should consider targeting visual perceptual processing. There is an interesting parallel in reading intervention. A recent remediation study on dyslexia showed that only 12 h of playing action video games improved children's reading speed, most likely due to enhanced visual attention (Franceschini et al., 2013).

### Conflict of interest statement

The authors declare that the research was conducted in the absence of any commercial or financial relationships that could be construed as a potential conflict of interest.
